# Addressing the primary care physician shortage in an evolving medical workforce

**DOI:** 10.1186/1755-7682-2-14

**Published:** 2009-05-05

**Authors:** Shaheen E Lakhan, Cyndi Laird

**Affiliations:** 1 Global Neuroscience Initiative Foundation, Los Angeles, California, USA

## Abstract

**Background:**

Primary care physicians have been shown to play an important role in the general health of the communities in which they serve. In spite of their importance, however, there has been a decrease in the number of physicians interested in pursuing primary care fields, while the proportion of specialists continues to increase. The prediction of an overall physician shortage only augments this issue in the US, where this uneven distribution is particularly evident. As such, serious effort to increase the number of practicing primary care physicians is both necessary and beneficial for meeting this country's health care needs.

**Discussion:**

There are several factors at play which contribute to the decrease in the number of practicing physicians in primary specialties. Lifestyle concerns, such as schedule and income, as well as the lack of prestige associated with this field seem to be among the most prevalent reasons cited for the diminishing interest. Multifaceted concerns such as these, however, are difficult to adequately invalidate; doing so would not only require a great deal research, but also a good deal of time – a resource which is in short supply given the current physician shortage being faced. Thus, a more immediate solution may lie in the increased recruitment and continued support of those individuals who are already associated with primary care service. This is particularly relevant given the Association of American Medical College's goal of increasing medical school enrollment by 15% over the next 10 years.

Several groups have been shown to be large contributors to primary care in the US. Here, we focus on three such groups: minority students, International Medical Graduates (IMGs) and Osteopathic Physicians (DOs). Although these groups are highly diverse individually, they all share the distinction of being underutilized in regard to the current primary care shortages faced. Thus, through more fully accentuating these resources, some of the problems being faced by this nation's healthcare industry may be ameliorated.

**Summary:**

To improve our nation's heath and healthcare, it is our opinion in this commentary that we must determine a comprehensive approach to increase the number of practicing physicians in primary care which may include minority and underserved medical student recruitment, and acceptance of international medical graduates and osteopathic physicians. Although overtime some of the more underlying causes of primary care under-representation must be addressed, these previous options may offer more immediate aid, while recognizing and augmenting populations who already contribute greatly to our nation's medical system.

## Background

The US is currently thought to be facing a physician shortage; a burden which is predicted to worsen over time [1]. Some estimates see a 20% deficit in the workforce, or about 200,000 physicians, occurring by the year 2020 or 2025 [1]. Contained within these breaches is a concomitant decrease in the proportion of primary care physicians (PCPs) within the medical workforce [2]. The field of primary care is among the most general in medical practices. Practitioners are responsible for addressing a varied majority of personal health care needs, developing a long-term relationship with patients, and practicing in the context of family and community [3]. Correlations linking superior overall health of communities better served by PCPs illustrate the importance of this discipline while also highlighting the potential damages of PCP shortages [3,4]. Many reasons have been cited for the decreased interest in the field of primary care; among the most prevalent are low specialty prestige, workload, and lower salaries [5,6]. Given this information, it seems clear that effort must be geared towards changing how primary care is viewed so that it reflects not only prestige, but also higher personal reward and respectable incomes, which may in turn bolster the number of interested students. This solution, however, ignores the population of students who currently chose to serve in primary care, and similar individuals who may be more likely to become involved in this field. Because minds are often harder to change than policies, a more immediate solution to this growing problem may lie in an enhanced recruitment of populations more likely to pursue primary care careers. Specifically, these include minority groups, international medical graduates (IMGs) and osteopathic physicians (DOs).

This aim of this commentary is to provide a short overview of the current and projected primary care provider shortage in the US and to briefly survey potential measures to counter this shortage that we believe are viable options.

## Discussion

### Medical Student Enrollment

Before focusing on specific groups, it should be noted that one measure is in place to help with the overall physician shortage in the US. Expressly, this is a new initiative to increase enrollment in medical schools by 15% over the next 10 years [7]. Medical schools plan to do this by gradually enlarging the size of classes accepted. Both the Association of American Medical Colleges (AAMC) and the Council on Graduate Medical Education (COGME) also have recommended that the number of residency training positions be increased [8]. Of course, simply increasing the total number of medical students only offers the potential for a marked increase in the number of PCPs in the US workforce. It is a promising solution for the issue of general physician shortages, but may fact lead to greater disparities between the number of PCPs and other specialist if the underlying causes of these differences are not addressed. Thus, again it appears as though one possibility to narrow the gap between specialist and PCPs lie not only in the enhancement of the overall number of students, but also in diversifying the types of students enrolled in the medical schools themselves.

### Minority Students

Historically, medical schools in the US have been dominated by largely white male populations [9]. It was not until the implementation of affirmative action programs in the early 1970s that efforts to increase diversity were sought by these institutions [10]. In addition to the obvious goal of increasing the number of minority physicians, race-based affirmative action was also initially realized to improve health care to the poor [10]. The fact that minority individuals had been shown to contribute more in rural and remote areas, as well as underserved poorer communities guided this choice. Importantly, it is these areas which suffer most markedly from inadequate primary care, as well [11,12]. Thus, it follows that minority students and those of low socioeconomic background have also been associated with a greater likelihood of service in primary care [12]. In spite of minority physicians' evident importance, however, the number of minority or low socioeconomic background individuals in medical schools is drastically low, making up only 6.4% of graduating physicians from allopathic medical schools in 2006 [13]. More disturbing is the fact that the percentage of minority students enrolled in public medical institutions has stagnated over time [10]. Part of the reason for this trend is the controversy associated with race-based admission programs and the subsequent actions taken to modify or remove race-based admission preferences [14]. A major example of this was seen after the *Hopwood *case in 1996 when both Texas and California included propositions limiting affirmative action in admission procedures. Although the *Hopwood *precedent was abrogated in 2003, it escalated the anti-affirmative action movement after 1996 which has aided in the continued discrepancy in population equality by race/ethnicity [15].

The current physician workforce is therefore not representative of these large populations who possess low socioeconomic status and diverse cultural backgrounds [16,17]. To improve the cultural competence of the physician workforce through diversity there should be an emphasis on recruiting more medical school applicants who are true representatives of these populations. Post-baccalaureate programs have proven in the past to be effective in aiding pre-medical students from underserved communities through the medical school application process while strengthening their qualifications [18]. Continuing support of such pipeline programs is invaluable to the improvement of the physician workforce.

### International Medical Graduates

Another group for which large contributions to primary care have been documented is IMGs [7]. Again, this seems to be especially true in rural and underserved areas [19]. If supply and demand trends stay about the same, it is predicted that by 2025 the number of IMGs in practice will increase by 102,000, a figure that could go a long way in easing the previously discussed shortages [7]. IMGs willingly fill many residency positions left vacant by US medical graduates (USMGs), particularly in lower-paying primary care specialties such as family medicine, internal medicine, and pediatrics [12]. This makes them an invaluable resource to the US, because studies also show that they tend to practice here in the US after residency training in areas similar to USMGs [7]. The acceptance of IMGs by the US medical community, however, has been mixed [20]. Although recognized as important contributors to many underserved communities, efforts have been made in the past to limit the arrival and residency of IMGs in the US [20]. Reasons for this have been hypothesized to center around competition perceived by USMG from the ever-increasing IMG force in the US, as well as the high cost of training non-citizens in US hospitals [20]. However, with the current shortages, high numbers of physicians is beneficial, especially since IMGs fill roles otherwise left vacant by USMGs. Accordingly, incentives both to ease the transition and better prepare IMGs for the US workforce should be enforced in order to augment the motivation students studying abroad may have to practice in the US. Examples of such incentives include "initiatives that encourage cultural pride and respect as well as support groups, international meals, cultural retreats, adjusted advising systems, and ongoing faculty reflection on treatment of IMGs" [21].

### Osteopathic Physicians

In addition to IMGs, another underutilized, but fast-growing group of physicians that are making meaningful contributions to the primary care workforce are doctors of osteopathic medicine (DOs). A contributing factor to the draw towards primary care is thought to be the philosophy that osteopathic physicians serve by (i.e. the focus on treating the patient as a whole), rather than concentrating on specific disease processes [22]. This falls in line with the responsibility of a PCP to develop a meaningful relationship with their patients due to the usually long term nature of their care. According to the American Osteopathic Association, DOs are one of the fastest growing segments of health care professionals in the US [22]. In 2004, over 40,000 DOs were estimated to be in active practice with 47% in the specialty of general and family medicine, 8% in internal medicine, and 4% are in pediatrics [23]. Additionally, trends in graduate medical education have suggested a rise in osteopathic trainees entering family medicine residency programs accredited by the Accreditation Council for Graduate Medical Education (ACGME), as well as an increase in the percentage of DOs in the primary care medical workforce of the future [23]. Notable, however, is the fact that far fewer osteopathic medical schools than allopathic medical schools exist in the US [24]. Thus, an increase in osteopathic medical schools would not only open up more positions for American undergraduates interested in pursuing a career in medicine, but would likely graduate a large number of physicians interested in primary care. Also, it has been shown that osteopathic medicine in general is underutilization by minority populations, probably because of the small percentage of minority individuals in the DO population of the US [23]. Hence, awareness of these alternative medical practitioners may further aid in the care given to underserved populations, as well as interest more minority students to pursue this line of work.

## Summary

In short, the current and predicted future physician shortage is an issue that must be addressed by policy makers, medical educators, and hospitals alike. A shortage of PCPs in particular has the potential to negatively impact our most vulnerable populations, such as the elderly and those dependent on community clinics [25]. Minority students have been highly associated with primary care roles, and through keeping programs such as affirmative action in place, this necessary diversity in US medical schools will continue to progress. Programs should also be put into place to promote the valuable resource of IMGs, and cultivate their skills to better serve patients here in the US. The growing number of DOs has the potential to make a large impact on the physician shortage, particularly if osteopathic medical schools are able to expand their numbers. There is no single solution to this multifactorial problem, but rather a series of steps that must be taken toward better serving the needs of physicians and their patients.

## Abbreviations

AAMC: Association of American Medical Colleges; COGME: Council on Graduate Medical Education; DO: doctor of osteopathic medicine; IMG: international medical graduate; USMG: US medical graduate.

## Competing interests

The authors declare that they have no competing interests.
